# The Trifurcated Anatomy of The Upper Trunk of The Brachial Plexus: Suprascapular Nerve, Posterior Division, and Anterior Division

**DOI:** 10.1055/s-0045-1814116

**Published:** 2025-12-30

**Authors:** Junot Hortêncio de Souza Neto, Bernardo Couto Neto, André Bastos Duarte Eiras, Renato Harley Santos Botelho, Lucas Gonçalves Daflon, Marco Aurélio Rodrigues da Fonseca Passos

**Affiliations:** 1 Hand Surgery and Microsurgery Department, Hospital Universitário Pedro Ernesto, Universidade do Estado do Rio de Janeiro, Rio de Janeiro, RJ, Brazil; 2 Hand Surgery Department, Hospital Naval Marcílio Dias, Marinha do Brasil, Rio de Janeiro, RJ, Brazil; 3 Orthopedics and Traumatology Department, Faculdade de Ciências Médicas, Universidade do Estado do Rio de Janeiro, Rio de Janeiro, RJ, Brazil; 4 Orthopedics and Traumatology Department, Faculdade de Medicina, Universidade Federal Fluminense, Niterói, RJ, Brazil; 5 Hand Surgery and Microsurgery Department, Faculdade de Ciências Médicas, Universidade do Estado do Rio de Janeiro, Rio de Janeiro, RJ, Brazil; 6 Hand Surgery Department, Instituto Nacional de Traumatologia e Ortopedia, Rio de Janeiro, RJ, Brazil; 7 Anatomy Department, Faculdade de Ciências Médicas, Universidade do Estado do Rio de Janeiro, Rio de Janeiro, RJ, Brazil

**Keywords:** adult, brachial plexus, cadaver, clavicle, adulto, cadáver, clavícula, plexo braquial

## Abstract

**Objective:**

To provide a comprehensive depiction of the anatomy of the upper trunk and its distal trifurcation through the dissection of brachial plexuses obtained from adult cadavers.

**Methods:**

We dissected 40 brachial plexuses from adult cadavers preserved using a unique formalin-based technique developed and used by the Anatomy Department of our institution. Bilateral dissection was performed on 20 cadavers placed in the dorsal decubitus position with accurate arm adduction. While the primary focus was on the upper trunk's anatomy, a thorough exploration of the entire brachial plexus was achieved through an extended incision.

**Results:**

The posterior division of the upper trunk was consistently located between the suprascapular nerve and the anterior division. In 22 (55%) of the dissected brachial plexuses, the suprascapular nerve originated from the proximal region of the posterior division of the upper trunk, immediately after its bifurcation. For the remaining 18 (45%), the suprascapular nerve originated directly from the upper trunk.

**Conclusion:**

The distal trifurcation of the upper trunk of the brachial plexus includes the suprascapular nerve and the posterior and anterior divisions of the upper trunk.

## Resumo

**Objetivo:**

Fornecer uma descrição abrangente da anatomia do tronco superior e de sua trifurcação distal por meio da dissecção de plexos braquiais obtidos de cadáveres adultos.

**Métodos:**

Foram dissecados 40 plexos braquiais de cadáveres adultos preservados por meio de uma técnica única, baseada em formalina, desenvolvida e utilizada pelo Departamento de Anatomia de nossa instituição. A dissecção bilateral foi realizada em 20 cadáveres, colocados em decúbito dorsal, com adução precisa do braço. Apesar de o foco principal ser a anatomia do tronco superior, uma exploração completa de todo o plexo braquial foi realizada mediante uma incisão prolongada.

**Resultados:**

A divisão posterior do tronco superior situou-se consistentemente entre o nervo supraescapular e a divisão anterior. Em 22 (55%) dos plexos braquiais dissecados, o nervo supraescapular originava-se da região proximal da divisão posterior do tronco superior, imediatamente após a bifurcação. Entre os 18 (45%) restantes, o nervo supraescapular originava-se diretamente do tronco superior.

**Conclusão:**

A trifurcação distal do tronco superior do plexo braquial inclui o nervo supraescapular e as divisões posterior e anterior do tronco superior.

## Introduction

The interscalene triangle is a conduit for the subclavian artery and brachial plexus, with the subclavian vein following an anterior course outside this triangular space. The vein is enveloped by a fascial sheath, comprising portions of the deep cervical and clavipectoral fascia. 1 Originating from the ventral branch of the inferior cervical spinal nerves and the first thoracic spinal nerve, the brachial plexus emerges from the spinal cord through ventral and dorsal roots and rootlets. As it exits the intervertebral foramen, the spinal nerve generates a recurrent meningeal branch known as *Luschka's nerve* , along with a dorsal branch responsible for the sensorimotor innervation of the posterior neck region. Furthermore, a ventral branch contributes to the constitution of the brachial plexus. 2 3 4

The brachial plexus may receive contributions from the fourth cervical spinal nerve (C4; prefixed) or the second thoracic spinal nerve (T2; postfixed). The literature indicates a considerable variability in the prevalence of C4 or T2 contributions, with 67 to 75% considered typical for brachial plexuses. Among these, 17.5 to 48% are classified as *prefixed* , and 2 to 7.5% are categorized as *postfixed* . 5 6 7 8 The upper trunk forms through the fusion of the roots from C5 to C6 the middle trunk consists exclusively of the C7 root, and the lower trunk, from the union of the roots from C8 to T1. Each trunk gives rise to anterior and posterior divisions. The posterior divisions converge to create the posterior cord. In contrast, the anterior divisions of the upper and middle trunk combine to form the lateral cord, while the anterior division of the lower trunk continues to develop the medial cord. 9 10 11 In cases of upper trunk paralysis or traumatic injuries, a potential reconstructive approach involves using the single available root to reinnervate the deltoid through the posterior division of the upper trunk. Nerve transfers can be employed to restore elbow flexion. However, placing nerve grafts incorrectly from the single available root into the anterior division of the upper trunk and using distal-nerve transfers for elbow flexion may lead to suboptimal deltoid reinnervation due to the absence of intraplexal axons in the posterior division. Understanding this is crucial to avoid grafting into the wrong division. 12

**Fig. 1:**
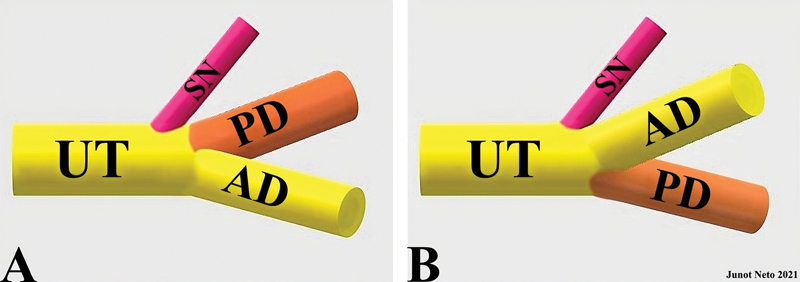
Upper segment of the brachial plexus ( **A** ) Anatomy of the upper trunk observed in cadaveric dissections. ( **B** ) Upper trunk anatomy commonly presented in various literature sources. **Abbreviations:** AD, anterior division; PD, posterior division; SN, suprascapular nerve; UT, upper trunk.

Describing the anatomy of the brachial plexus remains a formidable task, given its inherent complexity and the prevalence of anatomical variations. 3 9 In 1904, Dr. Wilfred Harris, a pioneering professor in the inaugural Neurology Department of a university hospital, authored an article titled “The True Form of the Brachial Plexus, and its Motor Distribution.” 13 He argued that the posterior division of the upper trunk of the brachial plexus consistently originated between the suprascapular nerve and the anterior division of the brachial plexus ( Fig. 1A ), contrary to most contemporary depictions and descriptions ( Fig. 1B ). 12 14 15 16 The existing incongruence in anatomical understanding prompted us to undertake the current cadaveric dissection study to elucidate the anatomy of the upper trunk of the brachial plexus and its divisions.

## Materials and Methods

The present study encompassed the dissection of 40 brachial plexuses from adult cadavers preserved using a light formalin-based technique developed and employed by the Anatomy Department of our institution. A bilateral examination was performed in 20 cases, all of male subjects aged 30 to 50. Cadavers showing any signs of injuries or suspected damage to the brachial plexus were intentionally excluded from the study. Fortunately, none of the dissected cadavers exhibited apparent brachial plexus injuries.

The dissections were conducted to elucidate the origins of the anterior and posterior divisions of the upper trunk of the brachial plexus and their relationship with the suprascapular nerve. The cadavers were positioned in dorsal decubitus with the arm adducted to maintain proper anatomical orientation. While the primary focus was on the anatomy of the upper trunk and its divisions, a comprehensive exposure of the entire brachial plexus in all investigated cadavers was achieved through an extended incision. This involved a longitudinal supraclavicular approach along the sternomastoid border, supplemented by a transverse supraclavicular incision over the clavicle, extending inferiorly along the deltopectoral groove.

The dissection of the supraclavicular plexus commenced with a deep incision in the skin, dividing the platysma. Subsequently, the supraclavicular nerves were identified beneath the platysma, and the deep fascia was incised along the specified line. In some instances, the fibers of the clavicular head of the sternomastoid muscle were dissected distally. Following this step, the omohyoid muscle was divided, and the fascial layer covering the scalene muscle was incised to expose the brachial plexus. An incision was meticulously made through the skin, subcutaneous tissue, and clavipectoral fascia to access the infraclavicular plexus, extending down to the clavicle. Subsequently, the tendon of the pectoralis minor muscle was located, elevated, and incised to reveal the complete length of the infraclavicular plexus. In every case, clavicle osteotomy was performed to optimize exposure. The anatomy of the upper trunk of the brachial plexus in all dissected cadavers was thoroughly documented using photographs, illustrations, and comprehensive written descriptions. These records were compiled for subsequent analysis.

The study was approved by the Plataforma Brasil ethics committee under CAAE number 95856618.6.0000.5259.

## Results

**Fig. 2:**
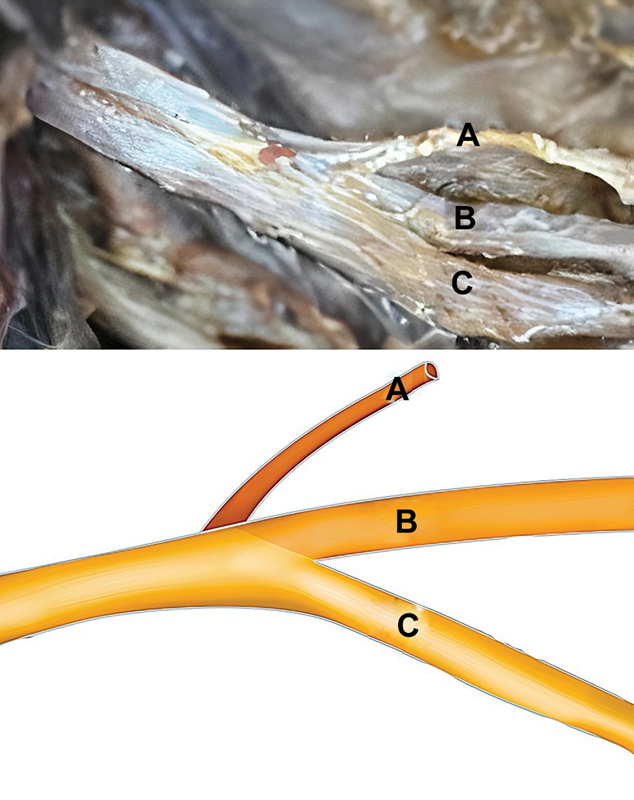
Upper-trunk anatomy based on cadaveric dissections. ( **A** ) The suprascapular nerve is positioned more cranially. ( **B** ) The posterior division originates cranially and dorsally, situated between the suprascapular nerve and the anterior division of the upper trunk. ( **C** ) The anterior division is the most caudal structure with a caudal and ventral origin.

A consistent anatomical pattern was observed across all 40 dissected brachial plexuses from the 20 cadavers. The posterior division of the upper trunk consistently occupied the most cranial position, originating cranially and dorsally just beneath the suprascapular nerve. Following this, the anterior division of the upper trunk assumed the most caudal position within the upper trunk of the brachial plexus, with caudal and ventral origins. Notably, the posterior division of the upper trunk was consistently located between the suprascapular nerve and the anterior division ( Fig. 2 ).

Additionally, the origin of the suprascapular nerve displayed two distinct patterns. In 22 out of the 40 dissected brachial plexuses (55%), the suprascapular nerve was found to emerge from the proximal region of the posterior division of the upper trunk, immediately after its bifurcation. Conversely, in the remaining 18 cases (45%), the suprascapular nerve originated directly from the upper trunk.

## Discussion

Describing the human brachial plexus through text or illustrations has proven to be an enduring challenge, despite centuries of anatomical studies. The intricacies arise from the complex interconnections within the plexus and the documented prevalence of anatomical variations in the literature. 3 9 Many authors replicate familiar brachial plexus illustrations without thoroughly examining the spatial relationships of their constituent structures0. Such descriptions often confine themselves to recognized interconnections, assuming these depictions provide a sufficient framework for understanding these connections. Hanna 14 points out that, for simplicity or convenience, the brachial plexus is frequently illustrated with the anterior division of the upper trunk positioned more cranially than the posterior division ( Fig. 1B ). Hanna 14 suggests that this convention likely dates to Andreas Vesalius, in 1555, and acknowledges that this portrayal might perpetuate a myth since, in practice, many peripheral nerve surgeons know that the accurate arrangement is not commonly publicized in most books and scientific articles ( Fig. 1B ).

Remarkably, Leonardo da Vinci, primarily renowned as a painter, made significant contributions to human anatomy during the sixteenth century. Under the guidance of Andrea del Verrocchio, Da Vinci embarked on anatomical endeavors involving the dissection of cadavers and the creation of numerous notes and illustrations. Da Vinci's proficiency in dissection, meticulous artistic renderings, and remarkable anatomical insights earned him acclaim. Among his notable achievements was the dissection and depiction of the brachial plexus. Interestingly, Da Vinci's illustrations portray the posterior division of the upper trunk with a cranial origin, while the anterior division of the upper trunk has a caudal origin 17 ( Fig. 1A ).

The initial acknowledgment of an inaccurate description of the anatomy of the brachial plexus can be traced to Harris, 13 in 1904, who concluded that the posterior division of the upper trunk had a cranial origin relative to the anterior division. He asserted that the posterior division consistently resided between the suprascapular nerve and the anterior division ( Fig. 1A ), emphasizing a deviation from the anatomy commonly published in the books and articles of his era ( Fig. 1B ). In 2016, Hanna 14 also stated this, highlighting the distal trifurcation of the upper trunk of the brachial plexus, arranged from cranial to caudal as the suprascapular nerve, the posterior division, and the anterior division. Hanna 14 termed this arrangement the *SPA arrangement* , in which ‘S’ denotes the suprascapular nerve, ‘P’ represents the posterior division, and ‘A’, the anterior division ( Fig. 1A ).

**Fig. 3:**
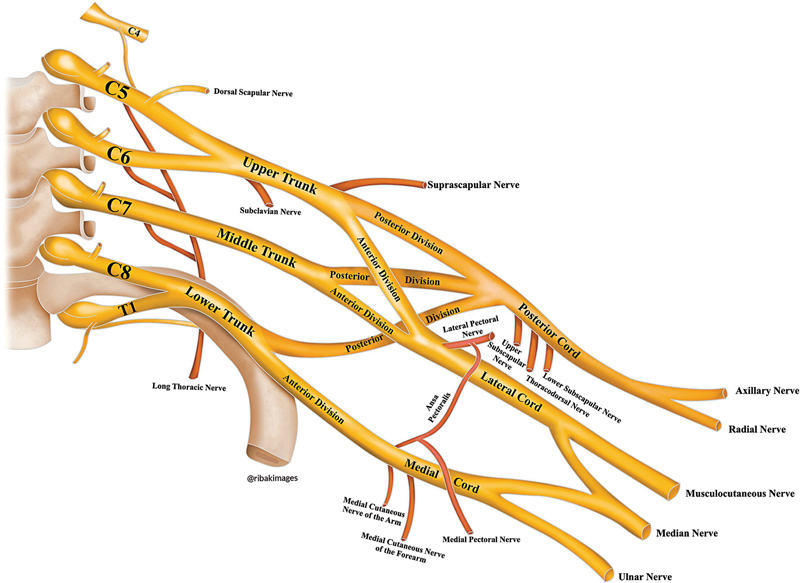
Illustration of the brachial plexus according to the findings proposed by Neto et al. 15

In 2015, Leung et al. 12 suggested that trauma might impact the arrangement of divisions within the upper trunk. To investigate this, they dissected 16 plexuses from fresh cadavers. Contrary to their initial hypothesis, Leung et al. 12 discovered that the suprascapular nerve consistently occupied the most lateral position within the upper trunk. Following the suprascapular nerve, the posterior and anterior divisions formed a trifurcation, with the posterior division consistently positioned between the suprascapular nerve and the anterior division ( Fig. 1A ). Neto et al. 15 reached a similar conclusion, observing that the upper trunk of the brachial plexus displayed a distal trifurcation. From cranial to caudal, the upper trunk included the suprascapular nerve and the posterior and anterior divisions. Once again, the posterior division consistently occupied the space between the suprascapular nerve and the anterior division ( Fig. 1A ). Neto et al. 15 illustrated a brachial plexus resembling the one shown in Fig. 3 .

Arad et al. 16 reported that, in 61% of dissected brachial plexuses, the suprascapular nerve originated from the posterior division of the upper trunk. In comparison, in 35% of the cases, it originated directly from the upper trunk. Only 4% of cases showed the suprascapular nerve originating from the root of C5. Notably, our findings revealed that the suprascapular nerve emerged from the posterior division immediately after its origin in 22 (55%) cases, with 18 (45%) originating directly from the upper trunk. None of the dissected plexuses had the suprascapular nerve arising from the root of C5. In alignment with Arad et al., 16 we agree that determining the precise origin of the suprascapular nerve can be challenging due to its proximity to the bifurcation point of the upper trunk and the multiple layers of mesoneurium surrounding the nerve. Importantly, when Arad et al. 16 concluded that the suprascapular nerve commonly originated from the posterior division, they acknowledged that the posterior division of the upper trunk of the brachial plexus had a cranial origin compared with its anterior division ( Fig. 1A ). This observation underscores that the upper trunk of the brachial plexus and its branches manifest differently in surgery than what is typically described in most anatomy books.

## Conclusion

The upper trunk of the brachial plexus displays a consistent and distinct distal trifurcation known as the *SPA arrangement* , as outlined by Hanna. 14 In this arrangement, from cranial to caudal, the upper trunk includes the suprascapular nerve and the posterior and anterior divisions of the upper trunk. Upon examination, 22 (55%) cases revealed the suprascapular nerve originating from the posterior division of the upper trunk, immediately after its origin. In the remaining 18 (45%) cases, the suprascapular nerve was found to derive directly from the upper trunk.
